# Study of the Activity of the *Staphylococcus aureus* Phage vB_SaS_GE1 Against *MRSA* Clinical Isolates and Its Impact on the Formation of Dual-Species Biofilms with *P. aeruginosa*

**DOI:** 10.3390/v17121623

**Published:** 2025-12-16

**Authors:** Nino Grdzelishvili, Davit Lazviashvili, Aleksandra Kurowska, Krzysztof Jakub Pawlik, Łukasz Łaczmanski, Elene Kakabadze, Elene Zhuravliova, Nina Chanishvili, Nata Bakuradze

**Affiliations:** 1 Laboratory of Microbial Biotechnology, George Eliava Institute of Bacteriophages, Microbiology and Virology, Tbilisi 0160, Georgia; davit.lazviashvili.1@iliauni.edu.ge (D.L.); nina.chanishvili@pha.ge (N.C.); 2 School of Natural Sciences and Medicine, Ilia State University, Tbilisi 0179, Georgia; ola.kurowska2@gmail.com (A.K.); elene_zhuravliova@iliauni.edu.ge (E.Z.); 3 Hirszfeld Institute of Immunology and Experimental Therapy, Polish Academy of Sciences, 53-114 Wroclaw, Poland; 4 David Tvildiani Medical University, Tbilisi 0159, Georgia

**Keywords:** bacteriophage, MRSA, *P. aeruginosa*, anti-biofilm activity

## Abstract

Bacteriophage therapy is regarded as a promising alternative for treating and preventing antibiotic-resistant bacterial infections. Methicillin-resistant *Staphylococcus aureus* (MRSA) is one of the most prevalent and difficult-to-treat pathogens. *S. aureus* also contributes to the formation of both single- and mixed-species biofilms. Treating biofilms remains a major challenge for antibiotic-based eradication of pathogens, as the biofilm matrix provides a protective barrier for bacteria. The selection of highly active phages targeting *S. aureus* is therefore crucial for medical applications, given the high prevalence and drug resistance of this pathogen. In this study, *S. aureus* phage vB_SaS_GE1 (GE1) was isolated and characterized as a potential therapeutic agent. The phage was isolated and propagated, and its host range was determined using standard methods. Whole-genome sequencing and annotation of the phage DNA were performed. A time–kill assay and evaluation of the anti-biofilm activity of the *Staphylococcus* phage, both alone and in combination with *Pseudomonas* phage GEC_PNG3 (PNG3) on mixed-species biofilms, were conducted. The results indicated that GE1 is a lytic phage that does not carry virulence-determining genes. The time–kill assay demonstrated sustained lytic activity of GE1 without the emergence of phage-resistant mutants in the tested MRSA strains. Although phage treatment increased biofilm matrix production compared to the control, the viable cell count within the biofilms was reduced. Overall, the characteristics assessed indicate that vB_SaS_GE1 is safe and exhibits strong antibacterial activity against MRSA strains.

## 1. Introduction

Bacteriophages, viruses that infect bacteria, are often recommended for treating bacterial infections that do not respond to conventional antibiotics. Depending on resistance properties and infection composition, phages may be administered individually, in mixtures known as phage cocktails, or in combination with antibiotics [1,2]. The demand for bacteriophage therapy has risen due to the global spread of antibiotic-resistant pathogens.

Among these, *Staphylococcus aureus* stands out as a major cause of severe and potentially lethal infections [1], including pneumonia, wound infections, sepsis, osteomyelitis and other conditions that often require hospital admission and long-term treatment. Both MRSA and methicillin-susceptible *Staphylococcus aureus* (MSSA) have contributed to an increasing epidemiological and economic burden in many countries [3].

In addition, *Staphylococcus aureus* is frequently detected as a co-infecting agent in mixed infections, such as chronic wound or respiratory infections, and it can be often underrepresented in biofilms formed by *Pseudomonas aeruginosa*. The formation of mixed-species biofilms by *P. aeruginosa* and *S. aureus* may render these communities up to 1000 times more resistant to antibiotics [2]. The complex nature of such infections slows down the treatment and therefore requires the development of new therapeutic approaches that would offer alternative solutions for eliminating resistant bacteria. Bacteriophage therapy has a long history of clinical use, particularly in countries of the post-Soviet Union, and it has recently been reintroduced worldwide as a promising solution for difficult-to-treat bacterial infections. Recent retrospective analyses of hundred clinical cases globally highlight the successful outcomes of phage therapy [4]. Growing evidence shows that phages can act synergistically with antibiotics—often reversing bacterial antibiotic resistance [5] or enhancing the antibacterial activity of the drugs [6]. Phages also contribute to biofilm degradation, both directly or by promoting antibiotic efficacy. Phage-derived hydrolytic enzymes such as depolymerases and lysins degrade the exopolysaccharide structure of biofilm, improving the penetration of the phage virions as well as antibiotics to reach the bacterial cells [3].

Despite the growing interest in therapeutic phages targeting *S. aureus*, only a limited number of well-characterized therapeutic candidates have been evaluated against MRSA in the context of mixed-species biofilms relevant to chronic wound infections. Existing phages such as Sb-1 and ISP have demonstrated promising activity [5,6], but there remains a need for additional strictly lytic, genomically safe phages with broad host range and documented performance in dual-species communities involving *P. aeruginosa*. In this study, we isolated and characterized the *S. aureus* phage vB_SaS_GE1 (GE1), assessed its genomic safety and taxonomic placement and evaluated its activity against MRSA isolates in both single- and mixed-species biofilms with *P. aeruginosa*. Our aim was to determine whether GE1 represents a suitable candidate for inclusion in phage-based strategies targeting biofilm-associated MRSA infections in chronic wounds.

## 2. Materials and Methods

### 2.1. Bacterial Strains

*Staphylococcus aureus* and *Pseudomonas aeruginosa* bacterial host strains used for the isolation and further propagation of bacteriophages were obtained from the collection of Eliava Institute of Bacteriophages, Microbiology and Virology (EIBMV). In total, 66 *S. aureus* clinical isolates were screened for susceptibility to phage GE1, of which 6 were identified as methicillin-resistant *S. aureus* (MRSA) and selected for detailed characterization, and one biofilm-forming *P. aeruginosa* clinical isolate (1147) was used in mixed-culture and biofilm experiments.

### 2.2. Antibiotic Susceptibility of Bacterial Strains

*Staphylococcus aureus* isolates from the Eliava Institute collection were tested for antibiotic resistance. A Kirby–Bauer antibiotic susceptibility test was used to select the methicillin-resistant *S. aureus* (MRSA) strains based on a cefoxitin susceptibility test. Briefly, freshly grown bacteria were grown on Muller–Hinton solid agar. Antibiotic disks were distributed over the bacterial lawn. After overnight incubation, inhibition zones were measured [7]. Interpretation of the results was based on the latest EUCAST manual (version 2024).

### 2.3. Bacterial DNA Extraction and MecA Gene Detection in MRSA Isolates

Methicillin-resistant *S. aureus* isolates were selected to be evaluated for the presence of the *mecA* gene. Freshly grown colonies grown on TSA agar were harvested and diluted in a lysis buffer. Bacterial DNA isolation was performed using an UltraClean^®^ Microbial DNA Isolation Kit (MO BIO, Carlsbad, CA, USA) following the manufacturer’s instructions. For the *mecA* gene detection, primers (F: AAAATCGATGGTAAAGGTTGGC R: AGTTCTGCAGTACCGGATTTGC) were selected based on the publication by J.H.Lee [8], and the related PCR steps were performed, respectively. To prepare 20 μL, mastermix AmpliTaq Gold^®^ Fast Master Mix (×1) at 10 μL, forward primer at 0.4–1 μL with final concentration of 0.2–0.5 μL/mL and reverse primer at 0.4–1 μL with final concentration of 0.2–0.5 μL/mL were used. The volume of 20 μL was filled with sterile DNA-se free water. The PCR amplification was performed with a hot-start, and primary denaturation was set at 95 °C for 10 min, which was followed by 40 cycles of denaturation at 94 °C for 30 s. Annealing was performed at 55 °C for 30 s and elongation at 72 °C for 1 min with a final elongation step at 72 °C for 5 min. For the detection of amplified gene fragments, gel electrophoresis was performed. A 1.5% agarose gel was prepared with 2.5 μL of ethidium bromide in 80 mL of agarose gel. Gel dye (6×) was diluted with PCR samples and run for 40 min at 80 v to detect 533 bp amplified gene fragments [8].

### 2.4. Isolation of the Bacteriophages Using the Enrichment Method

Isolation of the species-specific phages was performed using various sources, such as wastewater and clinical waste samples. The enrichment method was based on mixing 9 mL of potential phage source with 1 mL of 10× concentrated TSA broth and 1 mL of the bacterial culture. The mixture was incubated for 24 h aerobically at 37 °C. After incubation, the mixture was centrifuged at 6000 rpm for 30 min for pelleting bacterial debris. The supernatant was filtered through 0.20 µm pore filters [9].

### 2.5. Detection of Phages Active Against Target Pathogen and Their Propagation

Presence of species-specific phage virions in the obtained filtrates was checked using “the streak method”. The bacterial cultures were streaked on the TSA agar with a 10 µL loop and air-dried. A total of 10 µL of each filtrate was dropped on the surface of the streaks. The Petri dishes were incubated for 18–24 h at 37 °C to reveal the lytic zones on the grown streaks of the bacterial cultures. Clear lytic zones were cut out with a sterile loop and incubated in 2 ml of TSA broth for 2 h at 37 °C, to which 20 µL of chloroform was then added, thoroughly vortexed and stored at 4 °C at least for 30 min until further usage. For the propagation of the phages, the suspension was titrated using the double-layer agar (DLA) method. The bacterial isolates showing phage susceptibility were selected for further analysis of phage host ranges. Ten-fold serial dilution of the phage suspension was performed up to 10^−5^, and then 1 mL of each dilution was mixed with 100 µL of susceptible bacterial culture in the exponential growth phase, and a semisolid (0.6%) TSA agar warmed to 40 °C was added. The mixture was vortexed for a few seconds and applied on top of the solid TSA agar. The plates were air-dried and incubated for 18–24 h at 37 °C. After incubation, phage plaques were formed, and the lytic zones were evaluated according to their sizes and transparency indexes. To concentrate phages, several plates with the corresponding dilution showing a meshwork of plaques were prepared. The top layer from these plates were scraped off and collected in a 45 mL centrifuge tubes and centrifuged at 6000 rpm for 30 min. Afterwards, the supernatant was filtered to remove the bacterial debris, and the DLA method was performed to determine the concentration of the phage particles in the filtrate. Countable plates were used, and the number of plaques was multiplied by the dilution number and divided by the volume applied [9].

### 2.6. Study of the Phage Morphology

Transmission electron micrographs of the phages were obtained using JEOL-JEM-1400.

Phage concentrates with a titer of >2–5 × 10^10^ pfu/mL were used for the transmission electron microscopy (TEM) to study the morphology of the isolated phage virion, and the sample was prepared as described previously [10].

The size of the phages was calculated using the following formula:

*Size* [Angstrom (Å)] = *size of the image* [mm] × 10^7^
*magnification* (×220,000).

### 2.7. Phage Host Range and Efficiency of Plating (EOP)

Sixty-six bacterial isolates related to *Staphylococcus aureus* were used to study the phage host range. For this purpose, the streak method was applied. To evaluate the efficiency of plating, the phage was titrated and grown with each susceptible bacterial isolate using the DLA method. The efficiency of plating was measured by dividing the number of plaque-forming units (PFU) on target bacteria by its PFU on the host bacteria and was classified as given in Table 1 [11,12]. The isolates demonstrating high production (≥0.5) were selected as the host for further research.

### 2.8. Lytic Stability Assay

To assess the stability of GE1-mediated lysis, time–kill experiments were performed at different multiplicities of infection (MOI). Freshly grown MRSA cultures were adjusted 0.5 McFarland standard in TSA broth, to which phage GE1 was added to achieve MOI values of 1, 0.1 and 0.01, calculated as the ratio of plaque-forming units (PFU) to bacterial colony-forming units (CFU) at inoculation. The mixtures were incubated for 24 h to visualize the ability of the phage GE1 to suppress the growth of the target bacteria within a certain period of time. The incubation of the mixtures was performed for 24 h, and the bacterial growth was monitored at 3 h, 6 h, 18 h and 24 h [13].

### 2.9. Isolation of DNA for Whole-Genome Sequencing

The concentrate of the phage GE1, with a titer of ~3 × 10^10^ PFU/mL, was ultra-centrifuged at 21,000 rpm for 50 min to pellet the viral particles. The pellet was then resuspended in PBS and used for viral DNA isolation. To ensure samples remained uncontaminated with bacterial DNA and/or RNA in the subsequent experiments, we conducted the removal of bacterial genomic material. Phage lysates (450 μL) were treated with DNase I (1 U), RNase A (10 mg/mL) and 10× DNase buffer (50 μL) for 1.5 h at 37 °C to digest bacterial nucleic acids while preserving encapsulated phage genomes. Enzymes were inactivated with EDTA (20 mM), followed by capsid digestion using Proteinase K (1.25 μL; 20 mg/mL) for 1.5 h at 56 °C, without shaking [14]. To extract bacteriophage DNA, we used a DNA isolation kit (Qiagen DNeasy Blood *&* Tissue Kit, Hilden, Germany). Genome concentration was determined using a QuantiFluor^®^ dsDNA System on a Quantus™ Fluorometer following the manufacturer’s instructions.

Isolation and sequencing of the phage GEC_PNG3 (PNG3) DNA is described in a previous publication [15].

### 2.10. Whole-Genome Sequencing

Illumina sequencing libraries were prepared with a Nextera XT DNA Library Prep Kit following the manufacturer’s instructions [16]. Briefly, 150 ng of genomic DNA (5 ng/μL in 30 μL) was tagmented using Bead-Linked Transposomes at 55 °C for 15 min, the reaction was stopped with Tagment Stop Buffer at 37 °C for 15 min and the samples were washed three times with Tagment Wash Buffer on a magnetic stand. The tagmented DNA was then amplified using Enhanced PCR Mix with unique i7/i5 index adapters (5 μL each) under the following cycling: 68 °C for 3 min; 98 °C for 3 min; 5 cycles of 98 °C for 45 s, 62 °C for 30 s, 68 °C for 2 min; final extension at 68 °C for 1 min. Libraries were purified using Sample Purification Beads, quantified with a QuantiFluor^®^ dsDNA assay and assessed on a TapeStation 2200. Equal volumes of libraries were pooled, denatured (0.2 N NaOH), diluted to 6 pM, spiked with 1% PhiX and sequenced on a MiSeq Nano v2 300-cycle kit.

### 2.11. Genome Assembly and Annotation of the Phage GE1

Raw reads were quality-checked with FastQC v0.11.9, adapter- and quality-trimmed using Trimmomatic v0.32 (ILLUMINACLIP: NexteraPE-PE.fa:2:30:5, SLIDINGWINDOW: 4:25, MINLEN: 36) and assembled de novo using SPAdes v3.5.0 with default parameters. Annotation was performed using the PHROG database via the PHANOATE program. The visualization was prepared using the PHAROKKA program. The phage genomic DNA sequence was compared with other phage genomes using BLASTn +2.10 against the nucleotide database. Transfer RNA (tRNA)-encoding genes were identified using online tools tRNAscan-SE v2.0 (available at http://trna.ucsc.edu/tRNAscan-SE Access date: 10 June 2020) and ARAGORN v1.2.38. Virulence factors and antimicrobial resistance genes were screened with the EDGE v1.5 Gene Family module with default settings. Phage lifestyle was predicted with the DeepPL program. The presence of sequences associated with CRISPR-Cas systems was evaluated using CRISPRCas Finder (https://crisprcas.i2bc.paris-saclay.fr/. Access date: 20 October 2025) [17]. Potential anti-CRISPR protein (Arc) sequences were searched using the ArcHub server (https://pacrispr.erc.monash.edu/AcrHub/index.jsp Access date: 20 October 2025), which integrates three predictors: PaCRISPR, AcRankeri and an HMM-based predictor. Gene-family profiling (read- and contig-based) was performed against the Antibiotic Resistance Database, Resfams antibiotic resistance functions and Virulence Factors of Pathogenic Bacteria Database as well as the Comprehensive Antibiotic Resistance Database. Taxonomy of the phage GE1 was determined, and proteomic tree analysis was accomplished using ViPTree version 4.0.

### 2.12. Nucleotide Sequence Accession Number

The genome sequence for the phage GE1 was deposited into GenBank under the accession number OM030343.1.

### 2.13. Biofilm Formation Assay and Evaluation on Viable Cell Count

Multidrug-resistant *Staphylococcus* and *Pseudomonas* isolates were tested for biofilm production. Bacterial cultures freshly grown in TSA broth were diluted with 1% glucose containing TSA broth at a 1:1 ratio [18]. A total of 100 µL of each bacterial suspension was distributed to a 96-well plate in triplicate. The plate was incubated from 4 to 24 h at 37 °C temperature. After incubation, for phenotypical evaluation of the formed biofilms, a staining procedure was performed. First, the bacterial suspension was discarded to remove the planktonic cells, and the wells were gently washed twice with PBS. Afterwards, 125 µL of 0.1% crystal violet was added to the wells to stain the biofilms and incubated for 10–15 min at the room temperature. For the next step, the stain was washed with PBS 3 to 4 times and dried for several hours [18,19]. The optical density (OD) of the stained biofilms was measured with a Thermo Scientific™ Multiskan SkyHigh Microplate spectrophotometric reader at 570 nm. For the evaluation of the number of viable bacterial cells in the biofilms, after initial washing of the wells twice with PBS, 100 µL of PBS was used to remove the formed biofilms as a homogenized suspension and serially dilute them. A total of 10 µL of each dilution was distributed on TSA solid agar for the bacterial cell count to be performed [20].

### 2.14. Mono- and Combined Phage Activity Against Single and Mixed-Agent Biofilms

A total of 2–3 colonies of bacterial culture of each species freshly grown for 18 h were separately diluted in sterile saline solution and adjusted to 1.0 McFarland turbidity standard [21]. A total of 100 µL of each bacterial suspension was added separately to 100 µL of TSA broth containing 1% glucose for stimulating better biofilm formation by each bacterial culture [18]. To produce mixed biofilms, bacterial suspensions were combined with a ratio of 1:3 *v*/*v* of *P. aeruginosa* to *S. aureus*, and 100 µL of the mixed suspension was then added to 100 µL of 1% glucose TSA broth. A total of 200 µL of the received mixture was transferred in triplicate into 96-well plates and incubated for 24 h at 37 °C under static conditions. After incubation, wells with the grown biofilm were categorized into following experimental groups: (a) *Staphylococcus aureus* biofilms treated with GE1, (b) *Pseudomonas aeruginosa* biofilm treated with phage PNG3, (c) mixed biofilms treated with the phage cocktail (GE1 + PNG3) and (d) Sterile TSA broth (control). Old media was discarded from all wells, and the wells were gently washed twice with saline solution to remove planktonic bacterial cells [20]. Then, 200 µL of the phages GE1 and PNG3 diluted in TSA broth to a final titer of 3–5 × 10^6^ was added separately to their target bacterial biofilm groups, as well as in combination at a ratio of 1:1 *v*/*v* with the mixed-biofilm group, to receive MOI 0.1. An equal amount of sterile TSA broth was added to the control group. The plate was incubated for 18–24 h at 37 °C under static conditions. After incubation, antibiofilm activity was tested as described in a previous method by staining the wells with crystal violet and measuring the OD with a spectrophotometric reader at 570 nm. To measure the viable cell number, wells were gently washed twice with 200 µL of saline solution. The biofilm-entrapped cells were carefully collected by pipetting 200 µL of TSA broth and by simultaneously scraping off the biofilm with the tip of a pipette. From each well, 200 µL of biofilm was removed and placed in 1800 µL of saline solution, vortexed for homogenization and serially diluted to perform a viable bacterial count assay [22]. For counting the cells of each bacterial isolate, the following selective media were used: Pseudomonas-selective agar, and mannitol salt agar for cultivating the *S. aureus* cells [23].

Experiments assessing the impact of phages on biofilm formation were conducted in triplicate. The arithmetic mean of the triplicate measurements was calculated and used for data analysis. To confirm the robustness and reproducibility of the findings, independent experimental replicates (*n* ≥ 3) were performed.

### 2.15. Time–Kill Assay

The essential part of the study was to evaluate the ability of the phage GE1 to degrade single- and mixed-species infections. A clinical isolate from the bacterial strain collection *P. aeroginosa* 1147, on which the phage PNG3 demonstrated high lytic activity, was selected, along with two MRSA isolates (643 and 8497), to perform the time–kill assay. Bacterial distribution in a 96-well plate was performed similarly to the phage anti-biofilm activity assay described above. The phage solutions were applied in the same concentrations. A spectrophotometric reader was used to monitor the antibacterial activity of the phage solutions within 16 h at 600 nm [24,25].

## 3. Results

### 3.1. Antibiotic Susceptibility of Bacterial Strains

**Figure 1:**
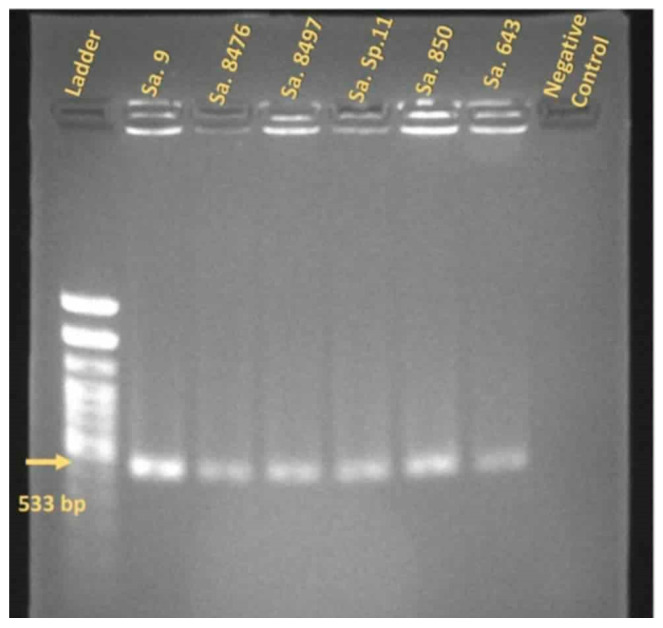
The PCR results of the MRSA strains that were positive for the *MecA* gene. Yellow arrow shows 533 bp gene fragment amplified in each sample. *E. faecalis* strain was used as a negative control.

First, the antibiotic susceptibility profiles and methicillin resistance status of the *S. aureus* isolates were determined to define the MRSA panel used for phage testing. Six methicillin-resistant *S. aureus* (MRSA) strains were selected out of 66 *S. aureus* isolates based on a Kirby–Bauer antibiotic susceptibility assay and *mecA* gene amplification tests. They were identified as MRSA based on phenotypical evaluation, showing resistance to cefoxitin (Table 2). The PCR revealed a 533 bp long fragment of the *mecA* gene (Figure 1) (Table 3).

### 3.2. Bacteriophage Isolation and Morphological Characterization

**Figure 2:**
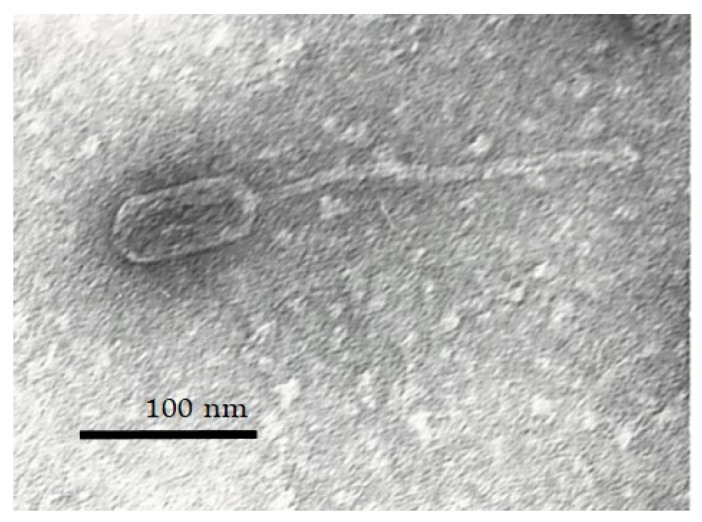
Transmission electron micrograph of phage GE1 at 220,000× magnification showing syphovirus morphotype.

Next, bacteriophage *S. aureus* vB_SaS_GE1 was isolated from the clinical waste of nasopharyngeal washing using an MSSA isolate as a host (*S. aureus* 152). The isolated phage produced 1 mm clear plaques on the agar plates. Electron microscopy of the GE1 phage exhibited typical characteristics of phages belonging to the *Herelleviridae* family, displaying an icosahedral head (72.7 nm) as well as a contractile tail (163 nm) and a baseplate attached to the tail (Figure 2). These observations are consistent with classification of GE1 as a lytic *S. aureus* phage and confirm its suitability for further genomic and functional analyses.

### 3.3. Study of the Phage Host Range and Efficiency of Plating

Having established the morphological features of GE1, we proceeded to evaluate its host range and efficiency of plating on *S. aureus* clinical isolates. A spot test assay was performed on 66 *S. aureus* strains, out of which 57 (86.3%) showed susceptibility according to the presence of the lytic plaques after phage application. A high productive infection (EOP ≥ 0.5) of the GE1 phage was evident for only 32.0% of the tested strains that previously have shown susceptibility results in the spot test. However, high productive infection together with the medium infection (EOP ≥ 0.1) of GE1 was shown for 70.0% out of susceptible *S. aureus* strains. Collectively, these data show that GE1 infects a high proportion of *S. aureus* isolates and achieves high or medium productivity in most susceptible strains, supporting its use as a broad anti-*Staphylococcus aureus* candidate.

### 3.4. Bacteriophage Genome Sequencing, Assembly and Annotation

**Figure 3:**
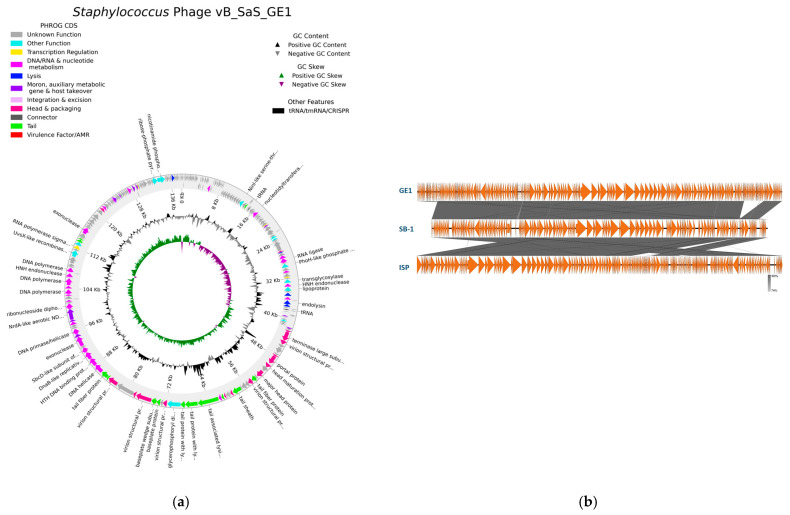
(**a**) Genomic map of vB_SaS_GE1; (**b**) comparative genomic analysis of GE1 with Sb-1 and ISP *Staphylococcus* phages.

The complete genome of bacteriophage GE1 was sequenced and annotated to assess its taxonomic position and to screen for virulence, lysogeny and antimicrobial resistance determinants. The complete genome of phage GE1 is 138,106 bp in size, has a GC content of 30.2% and contains 219 coding sequences (CDSs), of which 67 have assigned functional annotations, 41 are hypothetical proteins and 4 are tRNAs (Figure 3a, Table 4). No hits to known problematic virulence genes were detected at either the read or contig level. For comparative analysis, the GE1 genome sequence was queried by BLASTn, revealing 99.92% and 99.95% nucleotide identity to phages Sb-1 (NC_023009.1) and ISP (NC_047720.1), respectively, both of which are well-studied therapeutic phages [26,27]. Based on the BLASTn results, phage GE1 was assigned to the genus *Kayvirus*, within the subfamily *Twortvirinae* of the *Herelleviridae* family, representing a syphovirus morphotype. DeepPL analysis indicated that GE1 is a strictly lytic phage and lacks genes associated with lysogeny. CRISPRCasFinder identified only a single spacer and two direct repeat sequences; however, neither a leader sequence nor a cas gene cluster were detected. Out of the three algorithms used in the AcrHub ensemble, only PaCRISPR detected one putative anti-CRISPR protein, which overall does not indicate the presence of functional Arc proteins. Together, these genomic features indicate that GE1 is a strictly lytic *Kayvirus* lacking known virulence and resistance genes, which is desirable for therapeutic applications.

### 3.5. Antibacterial and Biofilm-Degrading Ability of the vB_SaS_GE1 Phage

**Figure 4:**
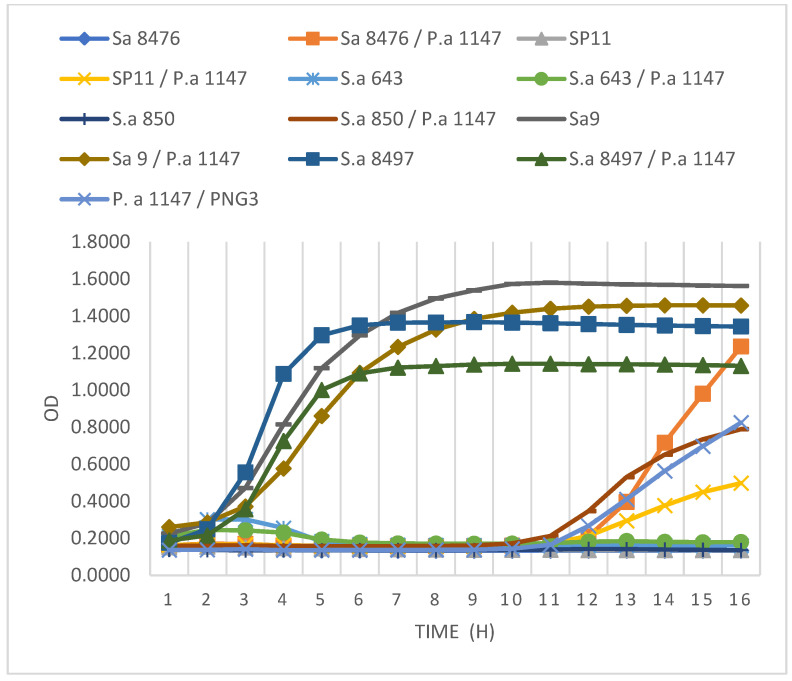
Results of the time–kill assay. MRSA strains grown as single and/or mixed cultures with *P. aeruginosa* strain after applying GE1 phage alone or in combination with pseudomonas phage PNG3.

Having confirmed that GE1 is a genomically safe, strictly lytic phage with broad activity against *S. aureus*, the antibacterial and anti-biofilm effects in single and mixed cultures of phage GE1 were evaluated. The activity of GE1 in combination with the PNG3 phage was studied in an 18 h time–kill assay on a single and mixed biofilms. Two of the six MRSA strains (*S. aureus* 9 and *S. aureus* 8497) showed no susceptibility to the GE1 phage, whether grown alone or in combination with *P. aeruginosa* 1147. The remaining MRSA strains showed suppressed growth in both single and mixed cultures. After 11 to 12 h, an increase in absorbance was observed, indicating renewed bacterial growth (Figure 4). A viable cell count assay on selective media demonstrated that this growth increase was due to the replication of PNG3-resistant *Pseudomonas* mutant forms. Overall, the time–kill assays showed that GE1 effectively suppressed the growth of GE1-susceptible MRSA strains in both single and mixed cultures, whereas the emergence of PNG3-resistant *P. aeruginosa* variants limited long-term control of the *Pseudomonas* population.

Based on the spot test and time–kill assay results, two MRSA strains were selected to evaluate the stability of GE1’s lytic activity over time: one fully susceptible strain (*S. aureus* 643) and one weakly susceptible strain (*S. aureus* 8497). *S. aureus* 643 showed no growth for 24 h at all tested MOIs. In contrast, *S. aureus* 8497’s growth was suppressed only at an MOI of 1 and only during the first 6 h.

**Figure 5:**
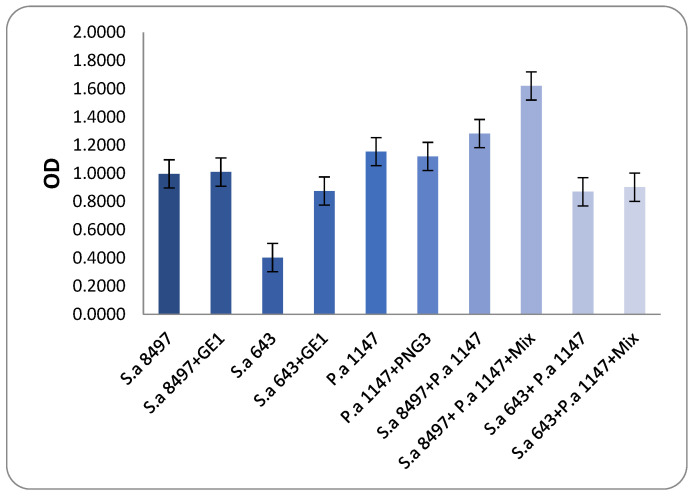
Biofilm-degrading assay. The results show that after phage application on developed biofilms, there was neither a substantial change nor an increase in biofilm production compared to the control groups. Mixture content—phages GE1 and PNG3.

**Figure 6:**
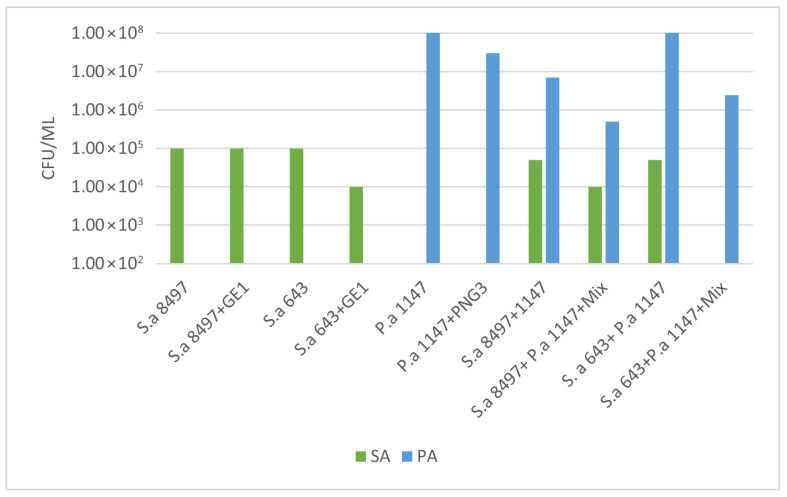
Bacterial count assay on the phage-treated biofilms. A 16 h coincubation of the single and mixed biofilms with the phage solution led to a decrease in *Staphylococcus* spp. bacterial counts both in the case of the single (*S. aureus* 643—fully susceptible to GE1) and mixed biofilms (*S. aureus* 8497 (weakly susceptible to GE1) + *P. aeruginosa* 1147). Mixture content: phages GE1 and PNG3.

These two MRSA strains were then combined with the biofilm-producing strain *P. aeroginosa* 1147. Viable cell counts and biofilm OD were measured to determine the anti-biofilm activity of phage GE1. In mixed biofilms formed by *S. aureus* 8497 and *P. aeruginosa* 1147, OD values did not change after phage treatment, but the viable counts of both species decreased by approximately 1 log (Figure 5 and Figure 6). No changes in OD or viable counts were observed when *S. aureus* 8497 was grown alone, confirming its resistance to GE1.

In contrast, when a mixed biofilm formed by *S. aureus* 643 and *P. aeruginosa* was treated with the phage solution, no viable *S. aureus* cells were detected, and *P. aeruginosa* counts decreased by 1 log. Notably, phage treatment increased biofilm formation in this experimental group. For biofilms formed solely by *S. aureus* 643, GE1 treatment did not alter OD, but the number of viable cells still decreased by 1 log compared with the control. In mixed biofilms formed by *S. aureus* 643 and *Pseudomonas*, biofilm density did not differ between treated and untreated groups, while viable counts of both *S. aureus* and *P. aeruginosa* were significantly reduced (Figure 5 and Figure 6).

In summary, phage treatment frequently reduced viable counts of MRSA and *P. aeruginosa* within immature biofilms, even when total biofilm biomass remained unchanged or increased, highlighting a dissociation between matrix production and cell viability under phage pressure.

## 4. Discussion

Bacteriophage therapy is emerging as a promising strategy against multidrug-resistant and biofilm-associated infections, where conventional antibiotics often fail. In this context, strictly lytic, genomically safe phages with broad activity against *Staphylococcus aureus*, including MRSA, and documented effects in mixed-species biofilms are of particular interest. This study positions GE1 as such a candidate, combining a favorable genomic profile with activity against MRSA in dual-species biofilms with *Pseudomonas aeruginosa*.

Lytic phages that lack virulence, lysogeny and antimicrobial resistance genes are widely regarded as the most suitable for therapeutic development [28,29]. GE1 fulfills these criteria and, in addition, is closely related to clinically explored phages Sb-1 and ISP at the genomic level [5,30], supporting its inclusion in the growing group of *S. aureus* phages with translational potential. The close relationship to Sb-1, together with shared overall genome organization and high nucleotide identity, suggests that GE1 may have comparable functional properties while still representing a distinct phage that can expand the available therapeutic repertoire.

An important implication of this work is that GE1 retains robust lytic activity against MRSA not only in planktonic cultures but also within early-stage mixed biofilms. The consistent reduction in viable MRSA counts, even when the total biofilm biomass remained unchanged or increased, underscores a mechanistic dissociation between matrix production and cell viability under phage pressure. This pattern aligns with the idea that phages can select for biofilm phenotypes while still effectively killing embedded cells, which has direct relevance for interpreting outcomes in biofilm-associated infections.

The observations in mixed cultures with *P. aeruginosa* highlight the complexity of polymicrobial infections. The emergence of PNG3-resistant *P. aeruginosa* variants, together with the modest reduction in a GE1-resistant *S. aureus* strain in mixed biofilms, suggests that interspecies interactions can modulate phage impact indirectly. The known ability of *P. aeruginosa* to alter *S. aureus* physiology and membrane composition may partially explain the increased vulnerability of otherwise phage- or drug-tolerant *S. aureus* subpopulations, pointing to a potentially exploitable synergy between community effects and targeted phage therapy [31].

The apparent increase in biofilm biomass after GE1 treatment, particularly with *S. aureus* 643, provides another mechanistic insight. Genomic predictions of peptidoglycan hydrolases and tail-associated proteins with potential depolymerase activity are consistent with phage-mediated remodeling of the biofilm environment, yet the net effect observed here was enhanced matrix accumulation alongside reduced viable counts. This supports the concept that, at least in immature biofilms, phage exposure can trigger a stress response in *S. aureus* that favors matrix overgrowth as a defense strategy, without fully preventing phage-mediated killing.

Several limitations of this work should be acknowledged. All experiments were performed in vitro using immature (24 h) biofilms and a limited number of MRSA isolates and a single *P. aeruginosa* strain, which may not fully capture the diversity and complexity of clinical infections. In addition, the study did not include in vivo efficacy or safety assessments, nor did it systematically evaluate combinations of GE1 with antibiotics commonly used to treat MRSA infections.

Despite these constraints, the data presented here demonstrate that GE1 is a strictly lytic *Kayvirus* with a favorable genomic safety profile, broad activity against *S. aureus* including MRSA and demonstrable efficacy in reducing viable bacterial counts in single- and dual-species biofilms. These properties support its potential as a component of phage-based strategies targeting biofilm-associated *S. aureus* infections, especially in polymicrobial contexts involving *P. aeruginosa*. Future work should extend these findings to mature biofilms and in vivo models, explore GE1 in combination with antibiotics and other phages and further dissect how interspecies interactions and phage-encoded enzymes shape biofilm architecture and treatment outcomes.

## Figures and Tables

**Table 1:** Classification of EOP.

The Average Efficiency of Plating Value	Classification	Characteristics
≥0.5	High production	The productiveness of infection of the target bacterial strain results in a PFU equal to 50.0% or more compared to the PFU found for the host.
0.1–0.5	Medium production	The productiveness of infection of the target bacterial strain is estimated between 10% and 50% of the PFU found for the host.
0.001–0.1	Low production	The productiveness of infection of the target bacterial strain is estimated between 1% and 0.01% of the PFU found for the host.
≤0.001	Inefficient production	Infection productiveness on the target strain is estimated below 0.001% of the PFU found for the primary bacterial host.

**Table 2:** Results of antibiotic susceptibility assay.

Bacterial Strains	Gentamicin	Erythromycin	Ampicillin	Lincomycin	Clindamycin	Cephalexin	Tobramycin	Oxacillin	Tetracycline	Cefoxitin
S.a 850	S	R	R	R	R	R	R	R	S	R
S.a 9	S	R	R	R	S	R	R	R	S	R
S.a 11	S	R	R	R	S	R	R	I	S	R
S.a 8497	S	S	R	S	S	S	S	S	R	R
S.a 8476	S	S	R	S	S	S	S	S	S	R
S.a 643	S	S	R	S	S	S	R	I	S	R

Note: Six MRSA strains are given, showing resistance towards cefoxitin.

**Table 3:** Susceptibility of MRSA strains to phage vB-SAS_GE1.

			Phage vB-SAS_GE1 Activity	
Isolate	MRSA	*MecA* Gene	10^8^ pfu/mL	10^7^ pfu/mL
S.a 9	+	+	1	0
S.a 8476	+	+	3	3
S.a 8497	+	+	1	0
S.a 11	+	+	4	3
S.a 850	+	+	5	4
S.a 643	+	+	5	4

Note: All six *S. aureus* strains identified as MRSA carried the *mecA* gene, but they showed variable susceptibility towards phage GE1. A grade of 5 indicates clear lytic activity, and a grade of 0 indicates no lytic activity.

**Table 4:** Genetic characteristics of vB_SaS_GE1.

Genome Size	GC%	CDS N	Genes with Functional Annotation N	Hypothetical Genes N	tRNA N
138,106 bp	30.2	219	67	41	4

## Data Availability

Data is contained within the article.
